# A Dynamic State‐Based Framework for Quantifying Triadic Autonomic Synchrony During Group Decision‐Making

**DOI:** 10.1111/psyp.70427

**Published:** 2026-10-16

**Authors:** Yubraj Gupta, Stefanie Suttkus, Uwe Altmann, Feliberto de la Cruz, Maria Geisler, Andy Schumann, Kar‐Jürgen Bär

**Affiliations:** ^1^ Department of Psychosomatic Medicine and Psychotherapy Jena University Hospital Jena Germany; ^2^ Department of Psychology MSB Medical School Berlin Berlin Germany

**Keywords:** autonomic signals, dynamic states, electrodermal activity, heart rate variability, interpersonal synchrony, *k*‐means clustering

## Abstract

In triadic social interaction, autonomic coordination is dynamic and may be poorly captured by session‐level synchrony estimates. We propose a state‐based framework to quantify time‐varying interpersonal synchrony during a laboratory group decision‐making task (Desert Survival Task) using concurrently recorded electrodermal activity (EDA) and ECG‐derived heart rate variability (HRV). Twelve triads (36 participants) with complete physiological recordings were analyzed. Signals were segmented into overlapping 60 s windows (1 s step). For each participant, window‐level features were extracted (EDA: 4 features; HRV: 74 features), standardized within individuals, and converted to dyadic divergence vectors (absolute differences) for the three dyads in each triad. Divergence vectors (combined EDA plus HRV) were reduced with PCA (95% variance retained) and clustered using a single global *k*‐means model trained on pooled windows to yield five discrete synchrony states shared across triads. States were ordered along the first principal component (PC1) of the divergence space, with its orientation chosen so that State 0 corresponded to the highest‐synchrony state (HSS). Triad‐level state metrics quantified both prevalence and temporal stability: fractional occupancy rate (FOR), mean dwell time (MDT), and conditional long‐run fraction (CLRF; ≥ 10 s runs). Empirical triads showed a graded continuum of synchrony across states and a strong bias toward the HSS. Surrogate validation using a leave‐one‐fixed Iterative Amplitude Adjusted Fourier Transform (IAAFT) null applied at the signal level demonstrated that HSS occupancy and stability exceeded chance alignment (FOR; *p* < 0.001; MDT and CLRF; *p* < 0.001). Convergent validity of the proposed state representation was further supported by continuous signal‐based benchmarks: compared with State 4, State 0 showed lower dynamic time warping distances and higher lag‐optimized physiological linkage (max *R* ^2^) for both EDA and HRV (all *p* < 0.001). In contrast, group‐mean anxiety measures (SPIN, STAI) were not reliably associated with HSS prevalence or stability in this sample. These findings suggest that discrete synchrony states capture interpretable, non‐random coordination regimes in triads and provide a complementary framework for characterizing the temporal organization of autonomic synchrony beyond conventional continuous synchrony measures.

## Abstract 2

We introduce a state‐based framework that converts triadic autonomic synchrony into a small set of recurring, comparable coordination regimes. Unlike session‐level averages, this approach quantifies how often groups enter high synchrony and how long they sustain it using occupancy and stability metrics. Validated against a leave‐one‐fixed IAAFT null and benchmarked with DTW and lag‐optimized physiological linkage, the method isolates non‐random coordination structure in group interaction and clarifies that trait‐synchrony associations may require larger triad samples.

## 1 Introduction

During social interaction, people often show coordinated patterns in their behavior and physiology. This phenomenon, commonly referred to as interpersonal synchrony, describes the temporal alignment of signals between interaction partners (daSilva and Wood 2025; Gifford et al. 2021; Novotny and Bente 2022; Tomashin et al. 2022; Yun et al. 2012). Synchrony has been observed across multiple systems, including body movement, autonomic physiology, and brain activity (Chidichimo et al. 2026; Gordon et al. 2025; Ohayon and Gordon 2025). In the autonomic domain, coordinated fluctuations in heart rate, heart rate variability (HRV), electrodermal activity (EDA), and respiration have been reported across a wide range of social contexts, such as cooperation, conversation, psychotherapy, and group decision‐making (Behrens et al. 2020; Gordon et al. 2020, 2021; Tschacher and Meier 2020).

Importantly, synchrony is not limited to dyadic interaction. In group settings, physiological alignment can emerge as a time‐varying network of pairwise relationships among multiple individuals, with coordination patterns changing across subgroups and moments of the interaction (Gordon et al. 2020; Wienhold et al. 2025). Group‐level physiological synchrony has been linked to collective experiences and outcomes such as cohesion and performance, motivating analytic approaches that can capture how coordination unfolds across multiple simultaneous interpersonal relationships (Gordon et al. 2020, 2021; Tomashin et al. 2022).

Interpersonal autonomic synchrony has frequently been associated with positive social outcomes, including rapport, empathy, trust, and perceived social connection (Gifford et al. 2021; Mayo et al. 2021; Palumbo et al. 2017; Tomashin et al. 2022). A common interpretation is that synchrony reflects shared emotional arousal or mutual engagement, supporting coordination and co‐regulation between partners. At the same time, synchrony is not uniformly positive (Wilson et al. 2018). Elevated physiological alignment has also been observed in stressful, conflictual, or high‐demand interactions (Gifford et al. 2021; Tomashin et al. 2022), suggesting that synchrony may reflect heightened vigilance or sensitivity rather than social closeness alone. These mixed findings highlight the need for models that capture how synchrony unfolds over time, rather than treating it as a single static quantity.

Despite growing interest in interpersonal synchrony, much of the existing literature relies on session‐level summary measures, such as mean cross‐correlations or coherence, or related indices computed across an entire interaction (Fu et al. 2021; Kleinbub et al. 2020; Tomashin et al. 2022; Wilson et al. 2018). While these approaches are useful for establishing whether synchrony is present overall, they cannot localize when synchrony increases or decreases, nor how coupling changes as the interaction evolves. This limitation is especially relevant in naturalistic or semi‐structured social settings, where emotional intensity, attention, and engagement fluctuate over time (Stoodley and Tsai 2021). Brief episodes of alignment may be diluted by averaging across long time periods, obscuring their psychological relevance. As a result, important information about the temporal structure of synchrony may be lost. To address these limitations, several studies have adopted time‐resolved approaches, such as sliding‐window cross‐correlation or windowed coherence analysis (Novotny and Bente 2022; Schoenherr et al. 2019). More generally, time‐resolved similarity can be quantified using metrics that allow temporal misalignment, such as dynamic time warping (DTW), which measures similarity under nonlinear temporal warping and has been used as a synchrony proxy in social and physiological sensing contexts (Gashi et al. 2019; Novotny and Bente 2022). In addition, interaction‐oriented approaches quantify lagged coupling by modeling one person's physiological variation as a function of a partner's responses over time (often referred to as physiological linkage; e.g., lagged regression/coupling quantified over a bounded lag range) (Helm et al. 2018; Thorson et al. 2021). However, time‐resolved estimates are often treated descriptively (e.g., as continuous curves) and do not explicitly test whether interactions revisit a small set of reproducible coordination modes. As a result, it remains unclear whether group interactions exhibit a limited number of recurring coordination regimes or fluctuate idiosyncratically from moment to moment. Critically, these approaches do not yield a small set of comparable coordination modes that can be summarized with interpretable temporal metrics (occupancy, dwell, transitions) and tested against null models.

An emerging alternative is to conceptualize synchrony as a sequence of discrete but recurring states, rather than as a continuously varying signal alone. A state‐based view directly tests whether interactions revisit a limited set of coordination regimes, and summarizes them with interpretable temporal metrics (e.g., occupancy, dwell time, transitions). In this framework, a synchrony state refers to a recurring pattern in a multivariate synchrony profile that can be identified using unsupervised state‐inference approaches, such as clustering or probabilistic state models (Li et al. 2021; Shine et al. 2016; Vidaurre et al. 2017). State‐based representations provide a compact description of high‐dimensional, time‐varying coordination and yield a small set of interpretable temporal metrics, including fractional occupancy, dwell time, and transition probabilities, that can be related directly to psychological variables (Lee et al. 2024; Vidaurre et al. 2017). Importantly, they also support stronger validity checks than descriptive time courses alone: apparent synchrony can be inflated by shared trends, autocorrelation, or other statistical structure unrelated to interpersonal coupling, making it essential to benchmark detected structure against appropriate null models (Altmann et al. 2022; Bernieri et al. 1988; Helm et al. 2018; Palumbo et al. 2017; Ramseyer and Tschacher 2010). Despite this potential, state‐based modeling has rarely been applied to interpersonal autonomic synchrony in triadic group interaction, and systematic validation of inferred synchrony states remains uncommon. Moreover, group studies face the additional challenge of defining states that are comparable across multiple triads, rather than yielding group‐specific labels.

Interpersonal synchrony may be particularly informative when examined in relation to individual differences. Social anxiety, characterized by heightened concern about social evaluation and increased self‐monitoring, has been linked to altered autonomic function during social interaction (Alvares et al. 2013). These alterations could plausibly shape how an individual's physiological signals align with others. Two competing hypotheses follow. Higher social anxiety may predict reduced synchrony, reflecting withdrawal or reduced social engagement. Alternatively, higher social anxiety may predict increased synchrony in certain moments, reflecting hyper‐attunement or heightened arousal under social stress. Recent work has reported that social anxiety can negatively predict physiological synchrony in dyadic joint action (Boukarras et al. 2025), underscoring the need to clarify when and how anxiety relates to physiological alignment, especially in group contexts.

In this exploratory study, we introduce a dynamic, state‐based framework for modeling interpersonal autonomic synchrony in triadic group interaction using EDA and ECG‐derived HRV. We (i) estimate time‐resolved synchrony from windowed multivariate dyadic divergence features, (ii) identify a small set of recurring synchrony states using a global clustering model that yields comparable state definitions across triads, and (iii) validate state structure against a leave‐one‐fixed Iterative Amplitude Adjusted Fourier Transform (IAAFT) surrogate null applied at the signal level, which preserves each individual's marginal distribution and power spectrum (approximate autocorrelation structure), while disrupting between‐person temporal alignment. We then test whether anxiety‐related traits are associated with the prevalence and temporal stability of the highest‐synchrony state. Finally, we assess convergent validity by benchmarking the highest‐ versus lowest‐synchrony states using continuous signal‐based metrics (DTW and lag‐optimized physiological linkage) computed in sliding windows and summarized at the triad level.

## 2 Methods

### 2.1 Data Source and Participants

The present study is a secondary analysis of an openly available dataset and its accompanying data descriptor (Burns et al. 2022; Gordon et al. 2021). Consequently, all physiological recordings were acquired according to the original experimental protocol described in these publications, and no modifications were made to the data acquisition procedures in the present study. The dataset, hosted on Mendeley Data (Burns et al. 2022), includes synchronized physiological recordings, video‐derived behavioral codes, and self‐report measures collected during a laboratory group decision‐making task. In the original study, 60 undergraduate participants (16 men; age: mean = 22.96 years, SD = 2.43, range 20–33) were assigned to 20 three‐person groups (triads). Triads were either all‐female or mixed gender. Individuals reporting heart conditions were excluded from participation in the original study (Gordon et al. 2021). All participants provided informed written consent, and the original protocol was approved by the Institutional Review Board of the Department of Psychology at Bar‐Ilan University (Burns et al. 2022; Gordon et al. 2021). The present reanalysis involves no additional data collection. We restricted our analysis to triads with complete EDA and ECG‐derived measures available for all three members during the group task. After applying this inclusion criterion, 12 triads (36 participants) were retained for analysis (Table 1). This conservative selection ensured that all synchrony measures could be computed consistently across all group members without the need for data imputation. Missing physiological data in the parent dataset primarily reflected recording artifacts or technical issues (especially for EDA), as documented in the data descriptor (Burns et al. 2022).

**TABLE 1:** Demographic characteristics of the selected participants (*n* = 36).

Characteristics	Value (mean ± SD)
Age	23.11 (2.71) years
Education	12.55 (1.41) years
Social anxiety scores (SPIN)	25.9 (14.04)
Trait anxiety scores (STAI)	70.9 (20.1)

### 2.2 Experimental Task and Procedure

Participants completed the Desert Survival Task (DST) (Lafferty and Pond 1974), a structured group decision‐making paradigm designed to elicit discussion, coordination, and joint problem solving. Following consent and questionnaire completion, participants were connected to physiological sensors and completed a 5‐min seated baseline period during which they were instructed not to move or talk (Burns et al. 2022; Gordon et al. 2021). The DST then began and was video recorded for subsequent behavioral coding. In the DST, group members first ranked 15 survival items individually and then discussed the items as a triad to reach a single consensus ranking (Burns et al. 2022; Gordon et al. 2021). The present analysis focuses exclusively on physiological dynamics during the group discussion phase, which involves spontaneous social interaction and coordination. Self‐report measures of anxiety‐related traits were collected prior to the DST using standardized questionnaires, including the State–Trait Anxiety Inventory (STAI) and the Social Phobia Inventory (SPIN). In the current study, SPIN and STAI scores were used to characterize triads via group‐mean anxiety levels in downstream analyses. Figure 1 provides an overview of the analysis pipeline from signal preprocessing and windowed feature extraction to global state inference.

**FIGURE 1:**
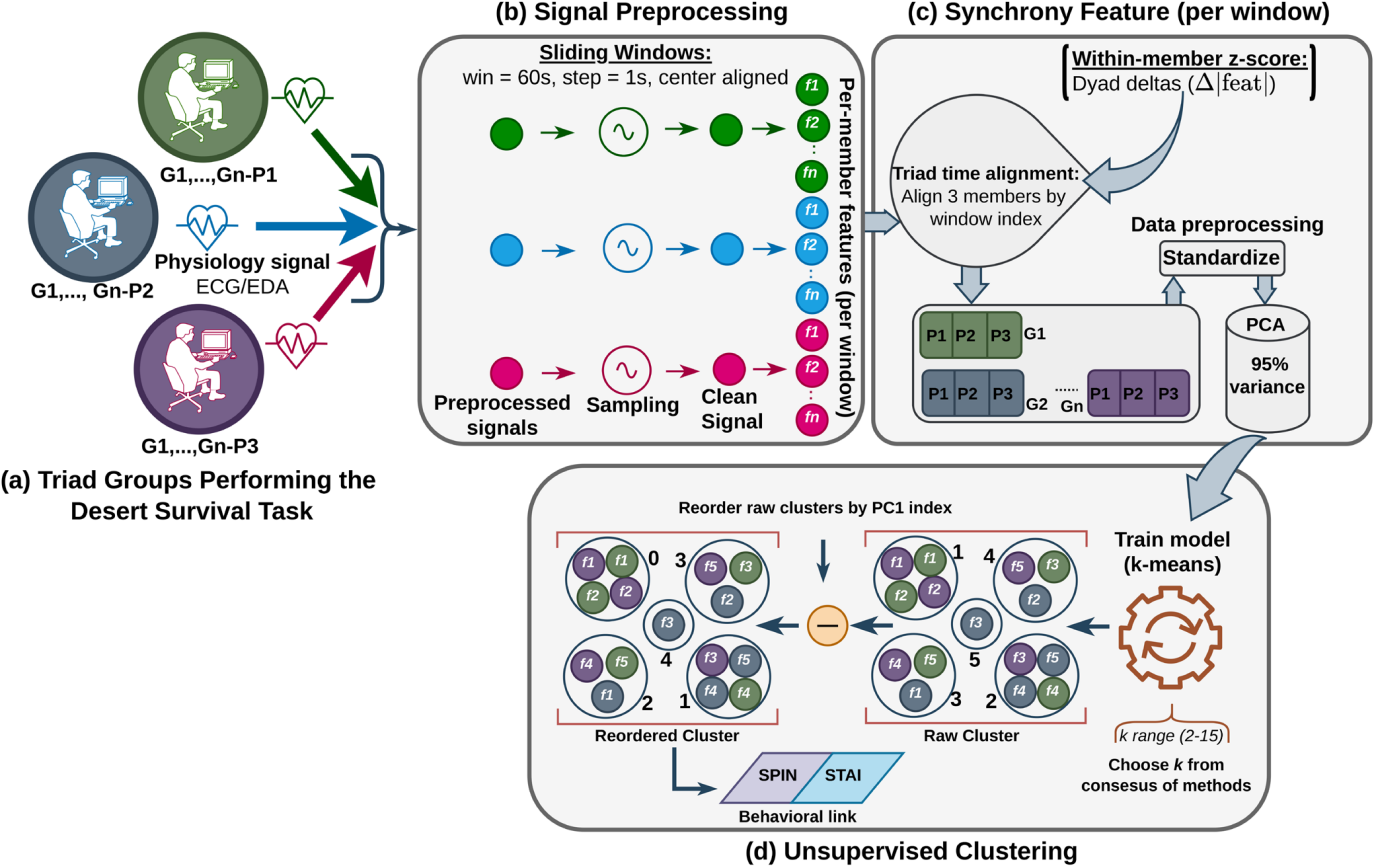
Overview of the dynamic state‐based pipeline for triadic autonomic synchrony. (a) Physiological recordings consist of triad groups (G1, …, Gn) with three participants per group (P1–P3) recorded while performing a desert survival task. ECG and EDA signals were acquired during the original experiment and subsequently underwent quality control and preprocessing by the dataset authors using the MindWare HRV and EDA software prior to data release (Burns et al. 2022; Gordon et al. 2021). (b) The present analysis begins with the cleaned preprocessed physiological signals provided in the public dataset. These cleaned recordings undergo additional processing and feature extraction, including reconstruction of HRV information from the cleaned IBI series, NeuroKit2‐based EDA processing, interpolation onto uniform time grids where required, and segmentation into overlapping, center‐aligned sliding windows (window length = 60 s; step = 1 s). Windowing is applied identically to all three members. (c) Synchrony features are constructed for each window. First, within‐member *z*‐standardization is applied across time to reduce individual baseline differences. Dyad‐wise divergence features are then computed as absolute differences between member pairs (AB, AC, BC), and a triad‐level summary (mean across the three dyads) is additionally computed for descriptive purposes. The three members are time‐aligned by window index. Divergence features are standardized and reduced using PCA, retaining components explaining 95% of the variance. (d) Unsupervised clustering is performed on the PCA‐reduced synchrony representation using a single global *k*‐means model trained on pooled windows across groups. The number of clusters is selected by consensus across multiple validity criteria over a candidate range (*k* = 2–15). Because raw cluster labels are arbitrary, clusters are reordered using the first principal component (PC1) of the divergence space. PC1 is oriented so that lower dyadic divergence (higher synchrony) corresponds to lower PC1 scores, and states are subsequently labeled in ascending order of their mean PC1, such that State 0 represents the highest‐synchrony state (HSS). The resulting state sequences are then used for downstream analyses, including associations with behavioral variables (e.g., SPIN, and STAI).

### 2.3 Physiological Data Acquisition

Physiological signals were recorded using MindWare mobile impedance cardiograph recorders (MindWare Technologies, Gahanna, OH) during baseline and the group task (Burns et al. 2022; Gordon et al. 2021). ECG and impedance cardiography were recorded at 1000 Hz. ECG recordings were acquired using the modified Lead II configuration reported in the original dataset acquisition protocol (Burns et al. 2022; Gordon et al. 2021). Respiratory and impedance cardiography signals were also recorded in the original protocol to support inter‐beat interval (IBI) related analyses. EDA was recorded using two disposable Ag‐AgCl electrodes placed on the palm of the nondominant hand. These signals were sampled at 500 Hz and transmitted wirelessly to the acquisition computer (Burns et al. 2022; Gordon et al. 2021). The dataset contains physiological recordings and behavioral measures (Burns et al. 2022).

### 2.4 Signal Preprocessing and Feature Extraction

Physiological signals released in the public dataset had already undergone quality control and preprocessing by the original authors using MindWare Technologies HRV and EDA software (v3.1.4) prior to data release (Burns et al. 2022; Gordon et al. 2021). ECG recordings were visually inspected, artifacts and ectopic beats were manually corrected, and inter‐beat interval (IBI) series were extracted. EDA recordings were visually inspected for corrupted segments, which were replaced using linear spline interpolation when appropriate, while recordings containing excessive artifacts (> 5%) were excluded from the original dataset. The present study therefore did not repeat the preprocessing performed by the original dataset authors but instead performed additional processing and feature extraction from the cleaned physiological signals provided in the dataset. To facilitate reproducibility, the complete analysis pipeline described in this section has been made publicly available in a GitHub repository (https://github.com/Yuvi‐416/dynamic‐triadic‐autonomic‐synchrony).

#### 2.4.1 HRV Feature Extraction

HRV analysis was performed from the cleaned IBI series provided in the original dataset rather than directly from the original ECG recordings. Although the original dataset had already undergone manual artifact correction using the MindWare HRV software (Burns et al. 2022; Gordon et al. 2021), an additional automated quality‐control procedure was applied to provide an additional, standardized quality‐control step prior to feature extraction. First, physiologically implausible IBIs (< 300 ms or > 2000 ms) were identified and excluded. The remaining intervals were then evaluated using a rolling median computed over the preceding seven valid IBIs. Intervals exhibiting a relative deviation greater than 25% from the local rolling median or successive interval changes exceeding 35% of the local rolling median were considered residual artifacts. Invalid intervals were subsequently replaced using linear interpolation to obtain a continuous IBI series while preserving the temporal structure of the recording. For each 60 s analysis window, the corrected IBI sequence was converted into R‐peak timing information compatible with NeuroKit2 (v0.2.5) (Makowski et al. 2021). HRV features were subsequently computed using the “*hrv_time”*, “*hrv_frequency”*, and “*hrv_nonlinear”* modules, yielding time‐domain, frequency‐domain, and non‐linear descriptors of autonomic regulation. In total, 74 HRV features were extracted from each analysis window to provide a comprehensive characterization of cardiac autonomic dynamics.

#### 2.4.2 EDA Feature Extraction

EDA feature extraction was performed from the cleaned skin conductance recordings supplied in the original dataset. The original recordings had already undergone visual quality control and artifact correction using the MindWare EDA software prior to release (Burns et al. 2022; Gordon et al. 2021). For the present analysis, the skin conductance signal was first linearly interpolated onto a uniform temporal grid with an effective sampling rate of 4 Hz to obtain equally spaced observations suitable for sliding‐window analysis. The uniformly sampled signal was subsequently processed using the default NeuroKit2 EDA pipeline. Because the interpolated signal had a sampling rate of 4 Hz, no additional low‐pass filtering was applied, consistent with the default NeuroKit2 implementation. The processed signal was decomposed into tonic skin conductance level (SCL) and phasic skin conductance response (SCR) components using the convex optimization (cvxEDA) algorithm, and SCR peaks were automatically identified using the NeuroKit2 peak detection procedure. Four descriptive features were then extracted from each 60 s analysis window: mean tonic level, tonic slope, phasic energy, and SCR rate.

#### 2.4.3 Sliding‐Window Feature Representations

Dynamic autonomic activity was characterized using overlapping 60 s windows with a 1 s step size, generating a continuous representation of physiological changes throughout the group interaction. A common window duration was adopted for both HRV and EDA to ensure temporal alignment between modalities, allowing physiological features extracted from both signals to correspond to the same time interval prior to multimodal integration. Although conventional HRV analyses commonly use longer recording segments, 60 s windows have been widely adopted in dynamic and ultra‐short‐term HRV analyses as a practical compromise between estimation stability and temporal resolution (Munoz et al. 2015; Shaffer and Ginsberg 2017; Laborde et al. 2017; Burma et al. 2021). This window length provides sufficient cardiac cycles for estimating the HRV features used in the present study while preserving the temporal resolution necessary to characterize evolving physiological interactions during group discussions. Using the same window duration for HRV and EDA ensured that synchronized multimodal feature vectors represented identical temporal epochs throughout the interaction, enabling direct integration of autonomic information across modalities without temporal misalignment. The 1 s step size enabled continuous tracking of evolving autonomic changes while maintaining substantial overlap between successive windows. For each valid window, the 74 HRV features and 4 EDA features were concatenated to form a 78‐dimensional physiological feature vector. Finally, all physiological features were z‐standardized within individuals to reduce inter‐individual baseline differences while preserving within‐person temporal fluctuations in autonomic activity.

### 2.5 Estimation of Interpersonal Synchrony

Interpersonal autonomic synchrony was quantified using combined (EDA + HRV) feature sets. All feature time series were first time‐aligned across the three participants within each triad at the window level, ensuring that each feature index corresponded to the same temporal segment for all members.

#### 2.5.1 Pairwise Feature Divergence

Let $x_{p,f}\left[t\right]$ denote the value of the feature *f* for the participant $\mathrm{p\epsilon}\left\{1,2,3\right\}$ at window *t*. To control for inter‐individual differences in baseline physiology and ensure that measures of divergence reflected temporal shifts in coordination, each participant's feature series was *z*‐standardized at the individual level across time:

(1) $$z_{p,f}\left[t\right]=\frac{x_{p,f}\left[t\right]-\mu_{p,f}}{\sigma_{p,f}},$$

where $\mu_{p,f}$ and $\sigma_{p,f}$ are the mean and standard deviation of $x_{p,f}\left[t\right]$ across all valid windows for that participant. For each window, pairwise divergence was computed as the absolute difference of *z*‐scored features:

(2) $$d_{\left(i,j\right),f}\left[t\right]=\left|Z_{\left(i,f\right)}\left[t\right]-Z_{\left(j,f\right)}\left[t\right]\right|,\left(i,j\right)\epsilon \left\{\left(1,2\right),\left(1,3\right),\left(2,3\right)\right\},$$

Smaller $d_{\left(i,j\right),f}\left[t\right]$ indicates greater similarity (stronger synchrony) for that feature at that time window.

#### 2.5.2 Triad‐Level Synchrony Representation

This procedure yields a multivariate, time‐resolved synchrony representation at each window consisting of pairwise divergence features. Because each triad comprises three dyads, the number of divergence features per window is fixed:

- **EDA**: 4 EDA features × 3 dyads = 12 divergence features/window.
- **HRV**: 74 HRV features × 3 dyads = 222 divergence feature/window.
- **Combined EDA + HRV**: (4 + 74) = 78 autonomic features × 3 dyads = 234 divergence features/window.

Consequently, every sliding window is represented by a feature vector with identical dimensionality and feature ordering. For example, in the combined (EDA + HRV) representation, feature positions (1–234) consistently correspond to the same physiological divergence measures (i.e., the same autonomic feature for the same dyad) throughout the interaction, while only their numerical values vary over time. This consistent feature ordering enables direct comparison of feature vectors across windows and supports subsequent multivariate analyses. In addition, for descriptive purposes, a triad‐level divergence summary (${△}_{f}\left[t\right]$) was obtained for each feature by averaging across the three dyads:

(3) $${△}_{f}\left[t\right]=\frac{1}{3}\sum_{\left(i,j\right)}d_{\left(i,j\right),f}\left[t\right],$$

where smaller ${△}_{f}\left[t\right]$ indicates stronger triadic synchrony for feature *f* at window *t*. All multivariate modeling and dimensionality reduction were performed on the full pairwise divergence representation (such as 234 features per window for combined analyses, respectively).

#### 2.5.3 Dimensionality Reduction

To address high dimensionality and redundancy, Principal Component Analysis (PCA) was applied to the standardized divergence features. It was applied after divergence computation to ensure that dimensionality reduction operated on synchrony‐related variability rather than individual physiological variance. Before PCA, divergence features were standardized across windows. Components explaining 95% of the total variance were retained. The retained PC scores were used as input to the global clustering model, and state ordering was defined using PC1.

### 2.6 Identification of Synchrony State

Recurring patterns of autonomic coordination were identified via unsupervised clustering of the PCA‐reduced trajectories. To ensure that state labels were directly comparable across triads, a single global model was trained on the pooled set of all valid windows from all empirical triads (rather than fitting separate clustering models per triad). Specifically, *k*‐means clustering was applied to the globally standardized dyadic PCA component scores at each window, partitioning windows into a small set of discrete synchrony states. This global state inference approach allows synchrony states to be defined consistently across triads, which has rarely been implemented in interpersonal synchrony research.

#### 2.6.1 Global Clustering Model

To identify coordination patterns that are comparable across the entire sample, window‐level PCA‐reduced feature vectors from all triads were concatenated into a single aggregate matrix. The optimal number of clusters (*k*) was evaluated on this pooled dataset over $\mathrm{k\epsilon}\left\{2,\ldots ,15\right\}$ using multiple cluster validity criteria (Elbow/inertia, Silhouette score, Calinski‐Harabasz index, Davies‐Bouldin index, and Gap statistic). The final *k* was selected by majority vote across these criteria, which indicated a *k* = 5 solution. The pooled matrix was then partitioned into five discrete synchrony states using the *k*‐means algorithm. By fitting the model to the pooled data, we ensured that the resulting state labels maintained a consistent physiological meaning across all triads and experimental conditions, enabling direct comparison of state‐based metrics between groups.

#### 2.6.2 State Ordering and Interpretation

Because *k*‐means cluster labels are arbitrary, states were re‐ordered using the first principal component (PC1) of the PCA‐reduced dyadic divergence space. PC1 was selected because it captured the largest share of variance in the divergence features and was negatively correlated with mean dyadic divergence, providing an interpretable ordering axis for synchrony. PC1 was oriented such that larger values corresponded to higher dyadic divergence (lower synchrony). States were then ranked by the within‐state mean PC1 score: State 0 was assigned to the lowest mean PC1 (highest synchrony), and higher state numbers corresponded to progressively higher mean PC1 (lower synchrony). This ordering was used for all downstream state‐based summaries.

#### 2.6.3 State‐Based Summary Metrics

For each triad, we computed state‐based metrics from the per‐window state sequence to quantify both the prevalence and temporal stability of synchrony states. Specifically, we derived (i) fractional occupancy rate (FOR; proportion of windows assigned to each state), (ii) mean dwell time (MDT; average length of contiguous runs in a state, reported in windows and converted to seconds using the step size), and (iii) conditional long‐run fraction (CLRF; proportion of runs in a state that exceeded a minimum run length). Given the 1 s step size, we defined a long run as a contiguous run of at least 10 consecutive windows (i.e., ≥ 10 s). The same global modeling procedure was applied to combined (EDA + HRV) divergence features to facilitate comparison across autonomic domains.

### 2.7 Validation With Surrogate Data

To test whether the empirical synchrony structure exceeded chance alignment, we generated surrogate triads using a leave‐one‐fixed strategy combined with Iterative Amplitude Adjusted Fourier Transform (IAAFT) surrogates applied at the signal level. Within each triad, one member's preprocessed (cleaned and uniformly sampled) EDA/HRV time series was kept unchanged, while the remaining two members' signals were replaced by independently generated IAAFT surrogates. IAAFT preserves each signal's amplitude distribution and power spectrum (and thus its autocorrelation structure) while disrupting the temporal alignment between members. Surrogate signals were subsequently processed using the same preprocessing, sliding window, and feature extraction pipeline as the empirical data to generate surrogate divergence feature vectors. Rather than estimating a new synchrony state model for each surrogate dataset, the surrogate‐derived feature vectors were processed using the empirical scaler and PCA projection before assignment to the nearest empirical *k*‐means centroid. This procedure isolates the effect of disrupting interpersonal temporal coordination while keeping the empirical synchrony state definitions unchanged. To ensure that the null distribution was not biased by any individual participant, the procedure was repeated three times for each triad, with each participant serving once as the fixed member while the remaining two participants were replaced by independently generated IAAFT surrogates.

#### 2.7.1 Fixed State Model Application

In this study, the term “fixed global state model” refers to the empirical synchrony state model consisting of the feature standardization parameters, PCA projection, and *k*‐means cluster centroids estimated from the original dataset. The empirical synchrony states were defined exclusively from the original dataset using the feature standardization parameters, PCA projection, and *k*‐means cluster centroids described in Section 2.6. During surrogate validation, these model parameters were not re‐estimated. Instead, surrogate‐derived feature vectors were transformed using the same scaler and PCA projection before being assigned to the nearest empirical cluster centroid:

(4) $$\hat{y_{s}}\left[t\right]=k‐{\text{means}}_{\text{global}}\left({\mathrm{PCA}}_{\text{global}}\left({\text{Scaler}}_{\text{global}}\left(X_{s}\left[t\right]\right)\right)\right),$$

where $X_{s}\left[t\right]$ denotes the surrogate‐derived divergence feature vector at window *t*, and $\hat{y_{s}}\left[t\right]$ is the synchrony state assigned by the empirical clustering model. Because the scaler, PCA projection, and cluster centroids remain fixed throughout the validation procedure, both empirical and surrogate data are evaluated within the same latent synchrony state space. Consequently, any differences in state occupancy or temporal organization can be attributed to disrupted interpersonal coordination rather than changes in the state definitions themselves. For each surrogate iteration, we computed the fractional occupancy of the highest‐synchrony state (HSS; State 0) to construct a null distribution against which the empirical HSS occupancy was compared. In addition to HSS fractional occupancy, we also evaluated HSS mean dwell time and conditional long‐run fraction.

### 2.8 Benchmarking Against Continuous Synchrony Metrics

To evaluate whether the discrete synchrony states identified by the proposed framework correspond to established continuous measures of interpersonal coordination, we benchmarked the highest‐synchrony state (HSS; State 0) against the lowest‐synchrony state (State 4) using two complementary continuous synchrony metrics computed directly from the cleaned preprocessed physiological signals: Dynamic Time Warping (DTW) and windowed physiological linkage. DTW quantifies waveform similarity while accommodating temporal delays between participants, whereas physiological linkage measures the strength of lag‐adjusted linear coupling between participants. Together, these metrics provide an independent validation of the proposed state‐based synchrony representation.

#### 2.8.1 Dynamic Time Warping (DTW)

For two discrete physiological time series within an analysis window, $X=\left(x_{1},x_{2},\ldots ,x_{N}\right)$ and $Y=\left(y_{1},y_{2},\ldots ,y_{N}\right)$ of length *N*, DTW computes the minimum cumulative alignment cost by allowing non‐linear stretching or compression along the temporal axis. The local distance between samples *i* and *j* is defined as $d\left(i,j\right)=\left|x_{i}-y_{j}\right|$, and the cumulative cost matrix $D\left(i,j\right)$ is computed recursively as,

(5) $$D\left(i,j\right)=d\left(i,j\right)+\min\left\{\begin{array}{c} D\left(i-1,j\right)\\ D\left(i,j-1\right)\\ D\left(i-1,j-1\right) \end{array}\right\},$$

subject to standard boundary conditions $D\left(0,0\right)=0$ and $D\left(i,0\right)=D\left(0,j\right)=\infty$. The overall DTW score for the window is given by the total cost $D\left(N,N\right)$. Unlike point‐to‐point Euclidean distance, DTW aligns similar physiological patterns even when they occur with short temporal delays between participants. Lower DTW values indicate greater similarity between physiological trajectories and stronger interpersonal synchrony.

#### 2.8.2 Physiological Linkage

Physiological linkage was quantified using lag‐optimized linear coupling within each sliding window. For a pair of participants with discrete physiological signals $x\left[k\right]$ and $y\left[k\right]$ (where $\mathrm{k\epsilon}\left\{1,\ldots ,N\right\}$ indexes samples within the window), linear regression models were evaluated across temporal lags within a bounded interval:

(6) $$y\left[k\right]=\beta_{0}+\beta_{1}x\left[k-\tau \right]+\varepsilon \left[k\right],$$

where $\tau \in \left[-\tau_{\max},\tau_{\max}\right]$ denotes the discrete temporal lag. For each tested lag τ, the coefficient of determination $R^{2}\left(\tau \right)$ was computed, and the physiological linkage score $L_{\text{link}}$ for that window was defined as,

(7) $$L_{\text{link}}=\max_{\tau \in \left[-\tau_{\max},\tau_{\max}\right]}R^{2}\left(\tau \right),$$

This lag optimization accounts for short response delays between participants while preserving the strength of their linear coupling. Higher linkage values indicate stronger physiological coordination.

Both DTW and physiological linkage were computed independently for each dyad (AB, AC, and BC) within every sliding window *t*. The corresponding triad‐level metric was obtained by averaging the three dyadic values,

(8) $$M_{\text{triad}}\left[t\right]=\frac{M_{\mathrm{AB}}\left[t\right]+M_{\mathrm{AC}}\left[t\right]+M_{\mathrm{BC}}\left[t\right]}{3},$$

where *M* denotes either the DTW distance or the physiological linkage score $L_{\text{link}}$. The lag maximum lag bound was set to $\tau_{\max}$ = 15 s for both EDA and HRV to comfortably encompass the slower temporal dynamics of electrodermal activity while remaining appropriate for heart‐rate variability.

### 2.9 Statistical Analysis

All statistical analyses were conducted at the triad level, with synchrony metrics derived from the global state model as described above. Individual SPIN scores were first averaged within each triad to obtain a single group‐level anxiety index, consistent with prior work using this dataset and with the group‐level nature of the synchrony measures. Primary dependent variables included state‐based synchrony metrics extracted from the empirical data: (i) fractional occupancy of each synchrony state, with a primary focus on the HSS, (ii) mean dwell duration within each state, and (iii) conditional long‐run fraction of time spent in each state. These metrics were computed for combined (EDA + HRV) analyses. Associations between synchrony metrics and social anxiety were assessed using Pearson correlations. This parametric approach was used to quantify linear associations between triad‐level anxiety and state‐based synchrony measures; given the small sample size, we report correlation coefficients alongside exact *p*‐values to allow transparent evaluation of effect magnitude and direction. To assess whether observed HSS occupancy exceeded what would be expected by chance, empirical values were compared against null distributions derived from leave‐one‐fixed IAAFT surrogate data. For each triad, the empirical HSS occupancy was evaluated relative to its corresponding surrogate distribution, and significance was determined using a two‐tailed threshold (*p* < 0.05), reflecting the directional hypothesis that genuine interpersonal coupling would increase time spent in the HSS. Group differences between State 0 and State 4 in DTW and physiological linkage were assessed using non‐parametric tests (Mann–Whitney *U*), given the non‐normality of window‐level metrics.

All analyses were executed using Python (v3.8) and R (v4.6.1), maintaining consistent preprocessing, modeling, and validation pipelines. Given the exploratory nature of the present study and the limited sample size, we interpret our results by emphasizing effect sizes and robust behavioral patterns rather than relying on binary significance testing.

## 3 Results

### 3.1 Discrete Dynamic Synchrony States Capture Graded Triadic Coordination

To characterize time‐varying coordination during group interactions, we analyzed triad physiological time series (EDA and HRV, Figure 2a) using a global clustering model trained across triads (Figure 2). The model identified five recurring synchrony states forming an ordered continuum from higher to lower coordination. State ordering was derived from the first principal component score (PC1) in the dyad‐divergence feature space, oriented such that lower PC1 values indicate lower divergence (higher synchrony). The ordered states corresponded to monotonic shifts in PC1 (Figure 2b, left), supporting the interpretation that the model captures graded coordination rather than a binary synchronous/asynchronous pattern.

**FIGURE 2:**
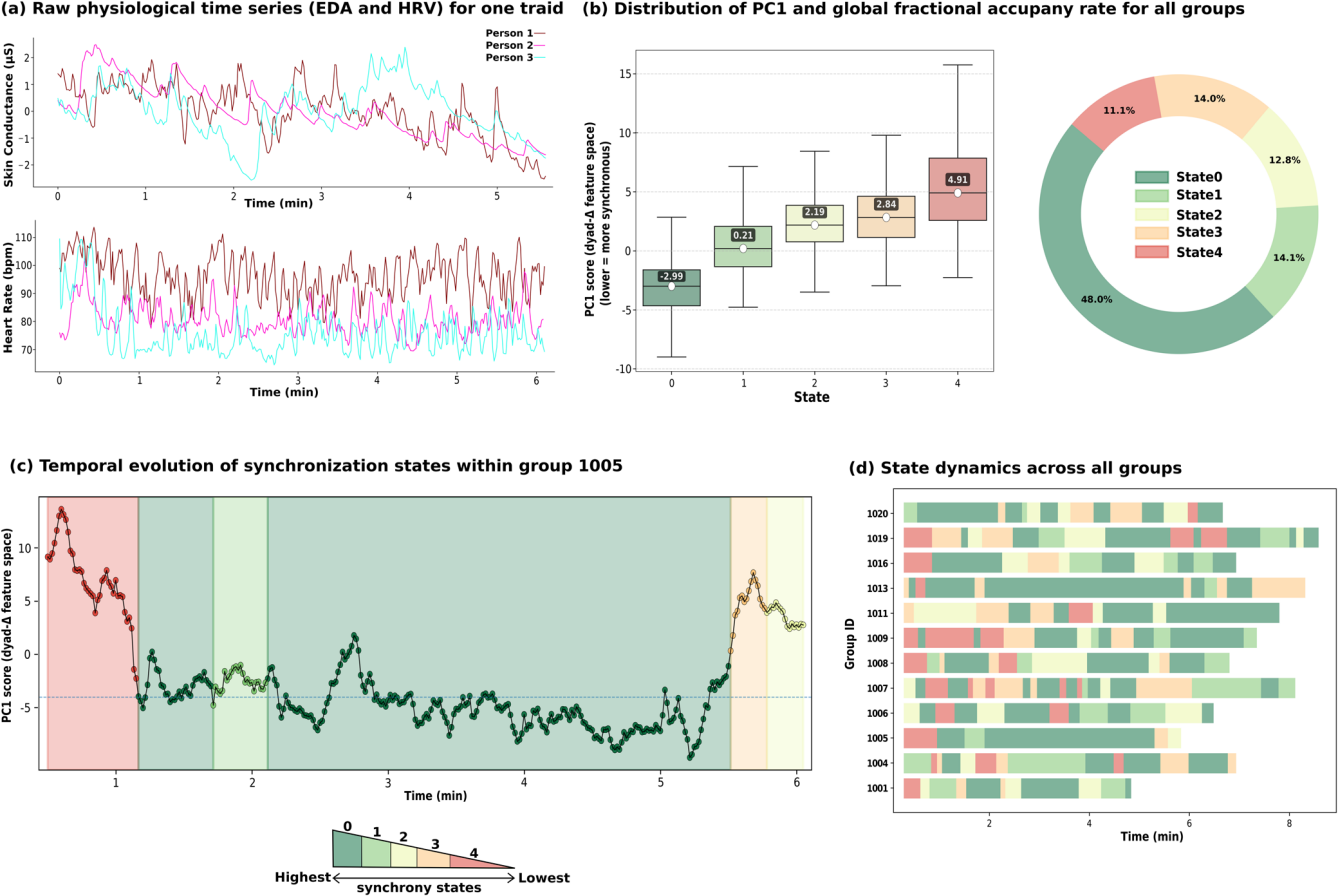
Discrete synchrony states derived from global clustering of triadic dyadic‐divergence features. (a) Example triad showing representative cleaned EDA and heart‐rate trajectories during the group interaction, illustrating within‐person variability and periods of co‐fluctuation across members. (b) State‐wise distribution of the first principal component score (PC1) computed in the dyad‐divergence feature space (lower values indicate higher synchrony) and pooled global fractional occupancy of each state across all retained triads. States are ordered by increasing PC1, with state 0 representing the highest‐synchrony state (HSS) and higher state numbers indicating progressively lower synchrony. (c) Within‐triad temporal evolution of the inferred state sequence alongside the PC1 trajectory, demonstrating switching between coordination regimes across time. (d) State sequences across all triads, showing between‐group heterogeneity in state persistence and switching while preserving a common global state definition.

HSS (State 0) showed the lowest PC1 values (median ≈ −2.99), whereas the lowest synchrony state (State 4) showed the highest PC1 values (median ≈ 4.91), indicating systematically increasing divergence across states.

At the sample level, State 0 was most prevalent. The pooled fractional occupancy (Figure 2b, right) showed that 48.0% of windows were assigned to State 0, with progressively lower occupancy for higher‐numbered (lower‐synchrony) states (State 1: 14.1%; State 2: 12.8%; State 3: 14.0%; State 4: 11.1%). State expression varied over time within triads. The example time course (Figure 2c; Group 1005) shows that the PC1 trajectory and discrete state labels change over the interaction, with both sustained runs and rapid switching, indicating that synchrony is dynamic rather than stationary. Extending this temporal analysis to the full cohort (Figure 2d), state sequences differed in both duration and switching frequency: some groups showed longer periods dominated by State 0, whereas others showed more frequent transitions and greater time spent in lower‐synchrony states. This variability underscores the value of modeling synchrony as a dynamic process rather than a static property.

### 3.2 Surrogate Validation Shows Increased Stability of the Highest‐Synchrony State in Empirical Triads

To test whether the highest‐synchrony configuration (State 0) reflects meaningful physiological coordination rather than random coincidence, we compared empirical triads against the leave‐one‐fixed IAAFT surrogate null (Figure 3). Across all three metrics, State 0 showed higher prevalence and greater temporal persistence in the empirical data than in the surrogates, indicating that the observed high‐synchrony dynamics are not readily reproduced when between‐person temporal alignment is disrupted. As illustrated in the fractional occupancy rate (FOR) analysis (Figure 3a), empirical groups occupied State 0 significantly more often than chance would predict (*d* = 4.46, *p* < 0.001). While surrogate triads did drift into this state randomly, the empirical data show a distinct and significant bias toward this high‐coordination configuration. Beyond simple occupancy, the temporal stability of these states provides the strongest evidence for genuine coordination. The mean dwell time in windows (MDT) analysis (Figure 3b) reveals that when real groups enter State 0, they sustain the state for significantly longer durations (*d* = 3.23, *p* < 0.001) compared to surrogates. Specifically, real groups maintained high synchrony for a median of approximately 13–25 time windows, whereas surrogates typically exited the state after only 4–8 windows. These findings are further reinforced by the conditional long‐run fraction (CLRF) metric (Figure 3c), which assesses the tendency of the system to remain in a state once established. Real groups exhibited a dramatically higher CLRF (*d* = 1.18, *p* < 0.001) with a median near 0.9, compared to approximately 0.60 for surrogates. Overall, the surrogate results show that disrupting interpersonal alignment reduces both the prevalence and persistence of the highest‐synchrony state, supporting the presence of structured, time‐dependent coordination in the empirical triads.

**FIGURE 3:**
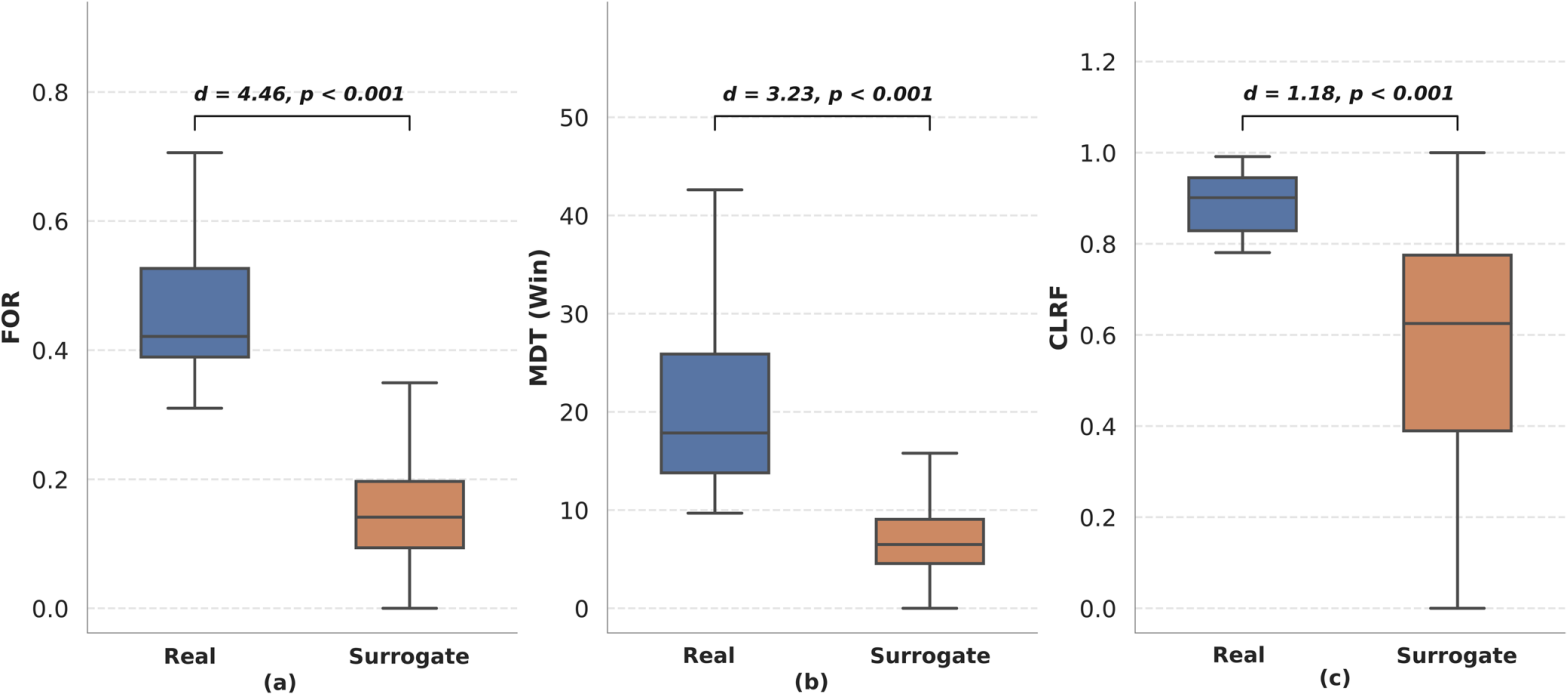
Surrogate validation of the highest‐synchrony state (State 0). Boxplots compare empirical triads (Real) against the leave‐one‐fixed IAAFT surrogate null (Surrogate) for three state‐based metrics: Fractional occupancy rate (FOR), mean dwell time in windows (MDT; Win), and conditional long‐run fraction (CLRF). Empirical data show higher State 0 occupancy (FOR; *d* = 4.46, *p* < 0.001) and substantially greater temporal stability (MDT and CLRF; both *p* < 0.001) than surrogates, indicating that the observed State 0 dynamics exceed chance alignment under the null.

### 3.3 Anxiety Traits Are Not Reliably Associated With State 0 Prevalence or Stability

To test whether the highest‐synchrony state (State 0) covaried with anxiety‐related traits, we computed Pearson correlations between triad‐level State 0 stability metrics (FOR, MDT, CLRF) and group‐mean psychometric scores (Figure 4). We did not observe reliable associations, as the observed relationships were weak and did not reach statistical significance in this sample. Confidence intervals are wide, consistent with limited power at the triad level (*n* = 12). For SPIN (Figure 4a), correlations with State 0 metrics were close to zero and slightly negative (FOR: *r* = −0.09, *p* = 0.792; MDT: *r* = −0.06, *p* = 0.856; CLRF: *r* = −0.07, *p* = 0.819). For STAI (Figure 4b), correlations were small and positive for MDT and CLRF but remained non‐significant (FOR: *r* = 0.02, *p* = 0.955; MDT: *r* = 0.25, *p* = 0.441; CLRF: *r* = 0.36, *p* = 0.256). These results suggest that, at the triad level, State 0 prevalence and stability were not strongly predicted by group‐mean anxiety scores in the current dataset.

**FIGURE 4:**
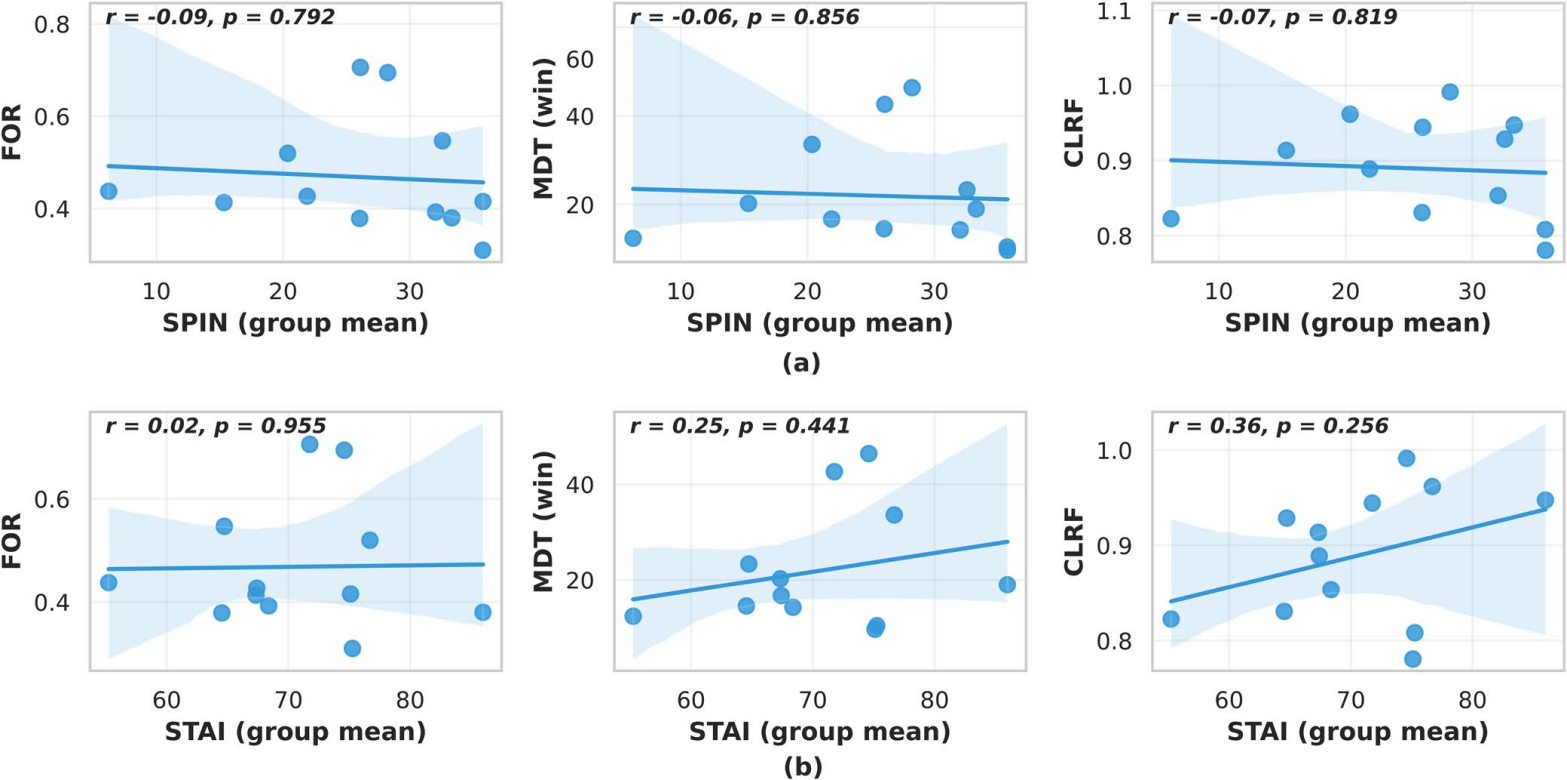
Association between high‐synchrony state stability metrics and group psychometric scores. Scatter plots show Pearson correlations between State 0 stability metrics (columns: Fractional occupancy, FOR; mean dwell time in windows, MDT; conditional long‐run fraction, CLRF) and group‐mean anxiety measures (top row: SPIN; bottom row: STAI). Each point represents one triad. Solid lines indicate linear regression fits with shaded 95% confidence intervals. In this sample, none of the associations reached statistical significance (all *p* > 0.05). SPIN showed near‐zero, slightly negative correlations with FOR, MDT, and CLRF, whereas STAI showed small positive correlations with MDT and CLRF and an approximately zero correlation with FOR.

### 3.4 Convergent Validity of Discrete States Using DTW and Physiological Linkage on Cleaned Physiological Signals

To assess whether the discrete synchrony states correspond to established continuous measures computed directly on the cleaned physiological signals, we compared the highest‐synchrony state (State 0) to the lowest‐synchrony state (State 4) using two windowed benchmarks: dynamic time warping (DTW) and physiological linkage (Figure 5). DTW quantifies similarity under non‐linear temporal warping, where lower values indicate greater similarity. Physiological linkage quantifies lag‐optimized linear coupling, defined as the maximum *R* ^2^ across a bounded lag range within each window, where higher values indicate stronger coupling. For visualization, values were summarized at the triad level per window (mean across dyads) and plotted as the mean across windows with 95% confidence intervals.

**FIGURE 5:**
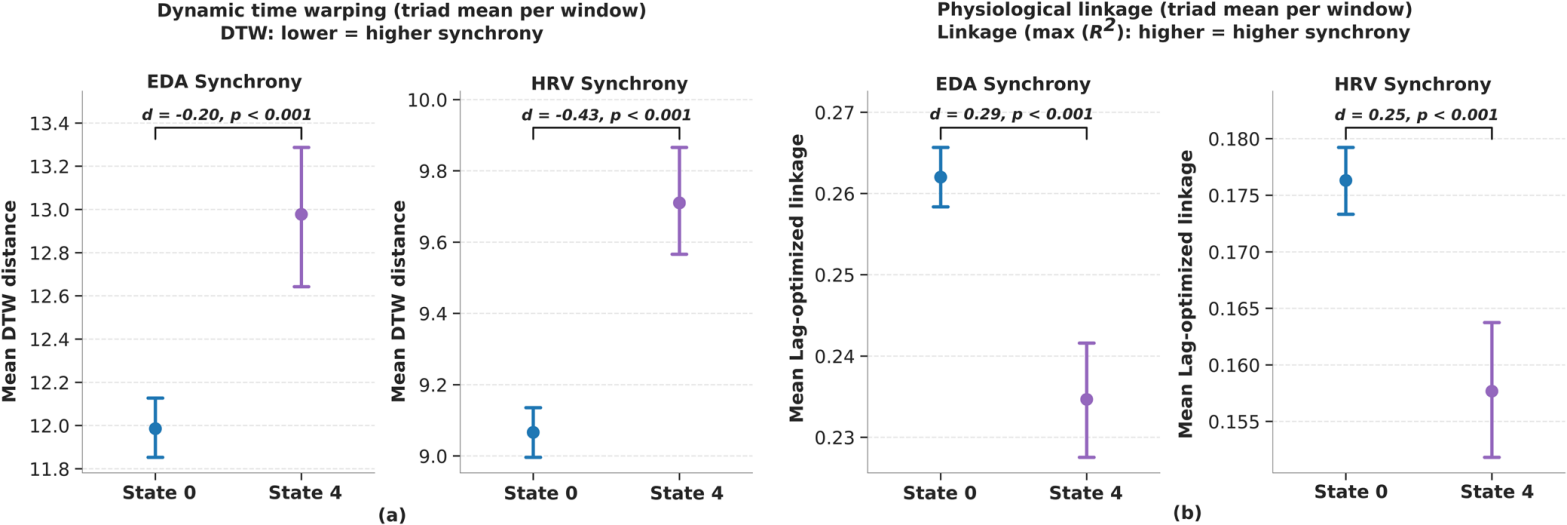
Convergent validity of discrete synchrony states using continuous signal‐based metrics (State 0 vs State 4). (a) Dynamic time warping (DTW) is computed on cleaned EDA (left) and HRV (right), summarized as triad‐mean DTW per window. Lower DTW indicates higher similarity. (b) Physiological linkage computed on cleaned EDA (left) and HRV (right), summarized as triad‐mean lag‐optimized linkage per window, defined as the maximum *R* ^2^ over a bounded lag range. Higher linkage indicates stronger coupling. Across both modalities and both benchmarks, State 0 shows higher synchrony than State 4 (DTW: Lower in State 0; linkage: Higher in State 0; all *p* < 0.001). Points indicate the mean across windows, and error bars denote 95% confidence intervals.

Across both modalities, the benchmarks aligned with the state ordering. As shown in Figure 5a, mean DTW was significantly lower in State 0 than in State 4 for both EDA and HRV (both *p* < 0.001, with *d* = −0.2 for EDA, *d* = −0.43 for HRV), indicating greater physiological‐signal similarity during the highest‐synchrony state. Similarly, Figure 5b shows that the mean lag‐optimized linkage was significantly higher in State 0 than in State 4 for both EDA and HRV (both *p* < 0.001, with *d* = 0.29 for EDA, *d* = 0.25 for HRV), indicating stronger linear coupling when the triad occupies the highest‐synchrony state. These results demonstrate convergent validity: the discrete state model's highest‐synchrony state corresponds to objectively higher similarity and coupling in cleaned physiological signals, while the lowest‐synchrony state corresponds to reduced similarity and coupling.

## 4 Discussion

In this study, we proposed a dynamic, state‐based framework to quantify triadic autonomic synchrony during an interactive group decision‐making task. EDA and ECG‐derived HRV features were extracted in overlapping 60 s windows with 1 s steps for each participant. Within each triad, features were time‐aligned at the window level, standardized within individuals, and transformed into dyad‐level divergence vectors (absolute differences for each pair). These divergence features provide a multivariate representation of within‐triad coordination at each time window. Dimensionality reduction (PCA retaining 95% variance) reduced redundancy, and a single global *k*‐means model trained on pooled windows identified five recurring synchrony states shared across triads. State labels were ordered by the first principal component (PC1) of the divergence feature space, oriented such that lower PC1 values reflect lower divergence (higher synchrony), and State 0 corresponded to the highest‐synchrony state (HSS). We then quantified state prevalence and stability via fractional occupancy (FOR), mean dwell time (MDT), and conditional long‐run fraction (CLRF). Empirical results showed (i) an ordered continuum of synchrony levels across states, (ii) non‐random prevalence and persistence of State 0 relative to a leave‐one‐fixed IAAFT surrogate null, and (iii) convergent validity of the discrete states with continuous signal‐based benchmarks (DTW and windowed physiological linkage). In contrast, triad‐level associations between HSS stability metrics and group‐mean anxiety‐related self‐report measures (SPIN and STAI) were not reliably detected in this sample.

### 4.1 Mechanistic Interpretation of Synchrony States

The inferred states can be interpreted as discrete coordination regimes that differ in the magnitude of multivariate dyadic divergence. Operationally, the state ordering reflects the PC1 axis of the dyadic divergence space, with lower PC1 indicating lower multivariate divergence (higher synchrony). By construction, lower divergence indicates greater similarity across participants' standardized physiological trajectories, consistent with tighter coupling in arousal‐related autonomic dynamics. Under this interpretation, State 0 reflects periods in which all three dyads within a triad exhibit relatively small divergence across the feature set, suggesting a coordinated autonomic response profile. Higher‐numbered states reflect increasing divergence, which may arise from asymmetric engagement, differences in cognitive load, or heterogeneous affective responses during the interaction. Importantly, the state representation does not assume that synchrony is constant throughout the interaction; rather, it models coordination as a sequence of regimes with variable duration and switching frequency. This aligns with interaction theories in which coordination is intermittently achieved and disrupted as group discussion progresses.

From a signal‐processing perspective, the divergence‐based construction captures similarity after removing individual baselines (within‐person standardization). Thus, state differences are driven primarily by shared within‐window fluctuations and relative changes, rather than stable trait‐like differences in absolute EDA or HRV levels. This improves interpretability: when State 0 occurs, it implies that members exhibit similar standardized trajectories within that window, whereas higher states indicate divergence in these trajectories. The presence of sustained runs in State 0 (as quantified by MDT and CLRF) further supports that high synchrony is expressed as temporally coherent episodes rather than isolated coincidences.

### 4.2 State‐Based Modeling Provides Information Beyond Session‐Level Metrics

A central motivation for this work is that session‐level synchrony estimates collapse temporal structure into a single value and can obscure meaningful dynamics. Two triads may show similar average synchrony but differ in how synchrony is organized over time (e.g., frequent short episodes versus fewer sustained episodes). The state‐based framework separates prevalence (FOR) from persistence (MDT, CLRF) and permits analysis of switching dynamics. In the current data, surrogate validation indicated that stability metrics, in particular, provided strong discrimination between empirical and null data, suggesting that temporal organization contains information beyond occupancy alone. This pattern supports the view that a key property of coordination is not only whether synchrony occurs, but whether triads can enter and maintain a coordinated regime.

The global modeling approach further improves comparability across triads. By training a single state model on pooled windows, state labels maintain a consistent meaning across groups, enabling direct between‐triad comparisons. This is difficult to achieve with per‐triad clustering or purely within‐dyad metrics, which can yield group‐specific scales that are not directly comparable. Moreover, by jointly representing the three dyadic relationships within each triad in a shared latent state space, the framework characterizes the overall configuration of autonomic coordination at the group level rather than interpreting each dyad in isolation. This common representation facilitates direct comparison of synchrony organization across different triads while preserving the temporal evolution of the interaction.

### 4.3 Convergence and Divergence Across EDA and HRV

EDA and HRV indices are complementary components of autonomic function and differ in temporal response properties. EDA reflects slower tonic dynamics and phasic responses, whereas HRV reflects beat‐to‐beat dynamics and autonomic regulation that may be sensitive to cognitive effort and regulatory demands. Despite these differences, the state framework provides a common representational space: both modalities can be mapped to dyadic divergence features, reduced via PCA, and clustered into ordered states. Convergent findings across modalities (e.g., lower DTW and higher physiological linkage in State 0 relative to State 4 for both EDA and HRV) support the interpretation that the inferred state ordering corresponds to measurable physiological‐signal similarity rather than a modality‐specific artifact.

At the same time, divergence between modalities is expected and potentially informative. EDA may emphasize shared arousal and engagement, whereas HRV may reflect cognitive or regulatory demands, leading to differences in the timing or strength of coordination episodes (Castaldo et al. 2019; Luque‐Casado et al. 2016; Pecchinenda 1996). A useful extension is to quantify cross‐modality coupling directly (e.g., whether transitions into high EDA synchrony co‐occur with transitions into high HRV synchrony) and to test whether one modality systematically leads the other during key interaction phases. This would help clarify whether the modalities reflect a single underlying coordination process or partially separable processes.

### 4.4 Discrete States Versus Continuous Synchrony Measures

A common concern with discrete‐state models is whether they introduce artificial boundaries in data that are fundamentally continuous. Here, the state model is not intended to replace continuous metrics but to provide a structured summary of time‐varying coordination. Benchmarking analyses address this concern directly: State 0 versus State 4 shows consistent separation on continuous measures computed on cleaned signals. DTW captures similarity under non‐linear temporal warping (lower values indicate higher similarity), whereas physiological linkage captures lag‐optimized linear coupling within each window (maximum R2 over a bounded lag range; higher values indicate stronger coupling). Agreement across both measures supports that the state ordering reflects a recognizable synchrony structure in the physiological recordings and is not solely an artifact of feature engineering or clustering.

State representation also offers practical advantages for interpretation and downstream analysis. Discrete states enable straightforward computation of FOR, MDT, CLRF, and transition‐based measures that are difficult to define consistently for continuous synchrony curves without introducing additional thresholding choices. States also facilitate group‐level comparisons and integration with behavioral and self‐report measures by providing a small set of interpretable variables.

### 4.5 Behavioral Relevance of High‐Synchrony Stability

We examined whether group‐mean anxiety‐related traits (SPIN and STAI) were associated with the prevalence or temporal stability of the highest‐synchrony state (State 0). We did not observe reliable associations as none of the correlations reached statistical significance in this sample, and effect sizes were small (Figure 4). SPIN showed near‐zero correlations with State 0 fractional occupancy, mean dwell time, and conditional long‐run fraction, whereas STAI showed small positive but non‐significant trends for mean dwell time and conditional long‐run fraction. These null findings should be interpreted cautiously. With only 12 triads, statistical power to detect moderate effects is limited, and triad‐level aggregation may obscure individual‐level or dyad‐specific effects. In addition, anxiety may relate more strongly to when synchrony increases (state transitions) or to asymmetric coupling patterns (e.g., leader–follower dynamics) than to overall time spent in State 0. Therefore, future work should test these possibilities using larger samples and multilevel models that retain individual and dyad‐level information while preserving a common state space across triads.

### 4.6 Limitations and Future Directions

Several limitations should be considered. First, the sample size at the group level was small (12 triads, 36 members), limiting statistical power and robustness of correlation estimates. Second, sliding windows are overlapping and therefore temporally dependent; the state model and stability metrics partially address this dependence, but future work could incorporate explicit time‐series state models (e.g., hidden Markov models) or block‐bootstrap procedures for inference. Third, the surrogate framework preserves univariate structure via IAAFT but may not capture all plausible null hypotheses (e.g., shared task‐driven responses). Alternative null models that preserve shared trends while disrupting interpersonal coupling would clarify which components of synchrony are interaction specific. Finally, while DTW and physiological linkage provide convergent validity, directional coupling approaches could test whether coordination is symmetric or whether leader‐follower dynamics emerge during discussion.

## 5 Conclusion

We introduced a dynamic, state‐based framework to quantify triadic autonomic synchrony during interactive group decision‐making using EDA and HRV. By converting individually standardized window‐level features into dyadic divergence vectors and applying PCA followed by a single global *k*‐means model, we identified five reproducible synchrony states shared across triads and ordered along a graded synchrony–desynchrony continuum using PC1 of the divergence space, with State 0 representing the highest‐synchrony state. Empirical triads spent a substantial proportion of time in the HSS and exhibited greater temporal stability of the HSS than expected under a leave‐one‐fixed IAAFT surrogate null, indicating non‐random, time‐organized coordination. Discrete state labels showed convergent validity with continuous signal‐based benchmarks: State 0 was characterized by lower DTW distances and higher lag‐optimized physiological linkage than State 4 in both modalities. In this sample, we did not detect reliable associations between triad‐level anxiety traits and HSS prevalence or stability, highlighting the need for larger group‐level samples and models that retain individual‐ and dyad‐level predictors. Overall, these results support discrete state modeling as a practical and interpretable approach for capturing the temporal structure of physiological coordination in triads, complementing conventional continuous synchrony metrics and enabling state‐based comparisons across groups.

## Author Contributions

**Feliberto de la Cruz:** methodology, supervision, funding acquisition, validation, writing – review and editing. **Maria Geisler:** methodology, validation, writing – review and editing. **Yubraj Gupta:** conceptualization, data curation, investigation, methodology, software, validation, formal analysis, visualization, writing – original draft, writing – review and editing. **Stefanie Suttkus:** conceptualization, methodology, validation, writing – review and editing. **Andy Schumann:** conceptualization, supervision, validation, project administration, funding acquisition, writing – review and editing. **Kar‐Jürgen Bär:** conceptualization, supervision, project administration, writing – review and editing. **Uwe Altmann:** methodology, validation, writing – review and editing.

## Funding

Andy Schumann and Feliberto de la Cruz received funding from the Interdisziplinäre Zentrum für Klinische Forschung (IZKF), Jena University Hospital, Germany (AMSP17, MSP19) and the German Research Foundation (SCHU3432/2‐1, CR 994/2‐1). The funding agencies had no role in the study design, the collection, analysis, or interpretation of data, the writing of the report, or the decision to submit the article for publication.

## Ethics Statement

The authors assert that all procedures contributing to this work comply with the ethical standards of the relevant national and institutional committees on human experimentation and with the Helsinki Declaration of 1975, as revised in 2008. All participants (and legal guardians for participants under 18) gave informed written consent.

## Conflicts of Interest

The authors declare no conflicts of interest.

## Data Availability

The data analyzed in this study are publicly available from Mendeley Data and the accompanying data descriptor (Burns et al. 2022; Gordon et al. 2021).
